# Changes in sex ratio from fertilization to birth in assisted-reproductive-treatment cycles

**DOI:** 10.1186/1477-7827-12-56

**Published:** 2014-06-23

**Authors:** Juan J Tarín, Miguel A García-Pérez, Carlos Hermenegildo, Antonio Cano

**Affiliations:** 1 Department of Functional Biology and Physical Anthropology, Faculty of Biological Sciences, University of Valencia, Burjassot, Valencia 46100, Spain; 2 Department of Genetics, Faculty of Biological Sciences, University of Valencia, Burjassot, Valencia 46100; and Research Unit-INCLIVA, Hospital Clínico de Valencia, Valencia 46010, Spain; 3 Department of Physiology, Faculty of Medicine, University of Valencia, Valencia 46010; and Research Unit-INCLIVA, Hospital Clínico de Valencia, Valencia 46010, Spain; 4 Department of Pediatrics, Obstetrics and Gynecology, Faculty of Medicine, University of Valencia, Valencia 46010; and Service of Obstetrics and Gynecology, University Hospital Dr. Peset, Valencia 46017, Spain

**Keywords:** Blastocyst-stage transfer, Cleavage-stage transfer, Preimplantation embryo development, Sex ratio, X-chromosome inactivation

## Abstract

**Background:**

In Western gender-neutral countries, the sex ratio at birth is estimated to be approximately 1.06. This ratio is lower than the estimated sex ratio at fertilization which ranges from 1.07 to 1.70 depending on the figures of sex ratio at birth and differential embryo/fetal mortality rates taken into account to perform these estimations. Likewise, little is known about the sex ratio at implantation in natural and assisted-reproduction-treatment (ART) cycles. In this bioessay, we aim to estimate the sex ratio at fertilization and implantation using data from embryos generated by standard in-vitro fertilization (IVF) or intracytoplasmic sperm injection (ICSI) in preimplantation genetic diagnosis cycles. Thereafter, we compare sex ratios at implantation and birth in cleavage- and blastocyst-stage-transfer cycles to propose molecular mechanisms accounting for differences in post-implantation male and female mortality and thereby variations in sex ratios at birth in ART cycles.

**Methods:**

A literature review based on publications up to December 2013 identified by PubMed database searches.

**Results:**

Sex ratio at both fertilization and implantation is estimated to be between 1.29 and 1.50 in IVF cycles and 1.07 in ICSI cycles. Compared with the estimated sex ratio at implantation, sex ratio at birth is lower in IVF cycles (1.03 after cleavage-stage transfer and 1.25 after blastocyst-stage transfer) but similar and close to unity in ICSI cycles (0.95 after cleavage-stage transfer and 1.04 after blastocyst-stage transfer).

**Conclusions:**

In-vitro-culture-induced precocious X-chromosome inactivation together with ICSI-induced decrease in number of trophectoderm cells in female blastocysts may account for preferential female mortality at early post-implantation stages and thereby variations in sex ratios at birth in ART cycles.

## Background

In Western gender-neutral countries, the sex ratio at birth is estimated to be ≈ 1.06 (for a review, see Hesketh and Xing [1]). This ratio is lower than the estimated sex ratio at fertilization which ranges from 1.07 to 1.70 depending on the figures of sex ratio at birth and differential embryo/fetal mortality rates taken into account to perform these estimations (for a review, see Pergament et al. [2]). Likewise, little is known about the sex ratio at implantation in natural and assisted-reproduction-treatment (ART) cycles. Nonetheless, implantation is a critical process that many embryos do not get through and, therefore, this event should be considered as important as fertilization or birth when analyzing changes in sex ratio through different stages of embryo/fetus development.

Fortunately, data from embryos generated by standard in-vitro fertilization (IVF) or intracytoplasmic sperm injection (ICSI) in preimplantation genetic diagnosis (PGD) cycles may be used to estimate not only the sex ratio at fertilization (primary sex ratio) in a more accurate way than previous studies (for a review, see Pergament et al. [2]) but also the still-unknown sex ratio at implantation. In this bioessay, we use data from IVF and ICSI embryos analyzed in PGD cycles as a proxy for estimating the sex ratio at both fertilization and implantation. Thereafter, we compare the sex ratios at implantation and birth (secondary sex ratio) in cleavage- and blastocyst-stage-transfer cycles to propose molecular mechanisms accounting for differences in post-implantation male and female mortality and thereby variations in sex ratios at birth in ART cycles.

## Methods

A literature review based on publications up to December 2013 identified by PubMed database searches using the following key words: sex ratio, preimplantation genetic diagnosis, cleavage-stage transfer, blastocyst-stage transfer, IVF, ICSI, biochemical pregnancy, fetal mortality, X-chromosome inactivation (XCI). This literature search retrieved a limited number of studies and put in evidence the absence of well-designed controlled randomized trials analyzing the concomitant effect of both insemination technique (IVF versus ICSI) and developmental stage at the time of embryo biopsy/transfer (cleavage versus blastocyst stage) on sex ratio of embryos/newborns. Notably, only one article [3] compiling the chromosomal sex of 117 IVF 4- to 8-cell embryos from PGD cycles was identified in our literature search. This is not surprising because during the early nineties, before the advent of ICSI, PGD technology was in its infancy, and patients and PGD laboratories were limited. For instance, the article by Griffin et al. [3] is a compendium of 27 PGD cycles performed in 4 separated series at the Hammersmith Hospital, London, over a 2-year period in 18 couples at risk of transmitting X-linked recessive disorders. Oocytes and embryos were cultured in Earle’s Balanced Salt Solution (EBSS) supplemented with 10% heat-inactivated maternal serum and biopsied blastomeres analyzed by fluorescent in situ hybridization (FISH). Consequently, estimates of sex ratios at fertilization and implantation based on data shown in Table 1 should be considered as relative values, not as absolute and precise figures. Estimates of sex ratios at birth from Table 2 are based on larger sample sizes and therefore are more robust than estimates of sex ratios at fertilization and implantation in IVF cycles. In any case, comparisons between groups in this bioessay should be performed in a qualitative way, not in a quantitative/statistical mode using meta-analysis or statistical inference methods.

**Table 1:** Sex ratio (XY/XX) of genetically diagnosed preimplantation embryos according to the method of fertilization applied and embryo developmental stage

**Method of fertilization**	**Four- to 8-cell embryos**^**a**^	**Day-5 blastocysts**	**References**
**IVF**	1.50^b^ (60/40)		[3]
	Total: 1.50^b^ (60/40)		
**ICSI**	0.88 (123/140)		[4]
		0.98 (225/229)	[30]
	1.18 (741/629)		[14]
	0.86 (96/112)		[7]
	Total: 1.09 (960/881)	Total: 0.98 (225/229)	

^a^In the IVF group, a total of 25 (17 males and 8 females) and 75 (43 males and 32 females) embryos were analyzed at the 4- and 8-cell stage, respectively. In the ICSI group, all the embryos were analyzed at the 8-cell stage.

^b^Sex ratio of 4- to 8-cell embryos would be 1.29 (66/51) if we consider 17 extra embryos that exhibited abnormal number of X and Y signals in the biopsied cell(s).

**Table 2:** Sex ratio (XY/XX) at birth of singleton deliveries according to the method of fertilization applied and the day of embryo transfer

**Method of fertilization**	**Day of embryo transfer**	**Sex ratio**	**References**^**a**^
**IVF**	≤ Day 3^b^	0.98 (1929/1968)	[23]
		1.08 (2084/1932)	[24]
	Total: ≤ day 3^b^	1.03 (4013/3900)	
	> Day 3^c^	1.22 (1030/846)	[23]
		1.28 (1088/852)	[24]
	Total: > day 3^c^	1.25 (2118/1698)	
**ICSI**	≤ Day 3^b^	0.94 (3047/3236)	[23]
		0.95 (2414/2542)	[24]
	Total: ≤ day 3^b^	0.95 (5461/5778)	
	> Day 3^c^	0.98 (1542/1566)	[23]
		1.10 (1289/1167)	[24]
	Total: > day 3^c^	1.04 (2831/2733)	

^a^Large-sample surveys using United States [23] and Australia and New Zealand [24] assisted reproductive databases.

^b^Cleavage-stage transfer.

^c^Blastocyst-stage transfer.

### Fertilization and preimplantation stages

It has been reported that human ejaculated spermatozoa display a normal Y:X ratio that does not differ from the Mendelian ratio [4-6]. Nevertheless, Table 1 shows that genetically diagnosed 4- to 8-cell IVF embryos exhibit sex ratios between 1.29 and 1.50. These figures contrast with the sex ratio closer to unity displayed by ICSI 8-cell embryos (1.09). Differences in sex ratios between IVF and ICSI embryos may be due to the fact that ICSI bypasses the zona pellucida and thereby any putative role it may have in selecting X- or Y-bearing spermatozoa (see below). Nevertheless, we should note that the sex ratio of cleavage-stage ICSI embryos is biased towards females when performing sperm selection for normal shaped nuclei, especially under high magnification (0.53, 112/210, in selected sperm injection versus 0.86, 96/112, in standard ICSI) [7] or when using the swim-up technique for preparation of spermatozoa from heavy smokers (0.47, 22/47, in heavy smokers; 0.95, 21/22, in slight-to-moderate smokers; and 1.13, 80/71, in non-smokers) [4].

There are several mechanisms that may account for the relatively elevated sex ratio found in IVF 4- to 8-cell embryos: (i) IVF male embryos may have a developmental advantage over female embryos after fertilization; (ii) the sperm preparation technique (either swim-up or three-layer discontinuous Percoll density gradient centrifugation) used in IVF may increase the proportion of Y-bearing spermatozoa; (iii) the molecular composition of the zona pellucida may render oocytes more susceptible to fertilization by Y-bearing spermatozoa; and/or (iv) Y-bearing spermatozoa may have higher fertilization ability.

Previous studies have reported that the sex ratio of preimplantation bovine embryos may be skewed towards males (i.e., preferential loss of female embryos) by manipulating the culture system including addition of glucose [8,9] and glucosamine [10]. In contrast, in humans the possibility that IVF male embryos have a developmental advantage over female embryos after fertilization is not supported by data on preimplantation embryo development. Firstly, it is known that ≈ 10% of all human IVF (or ICSI) embryos undergo early developmental arrest [11]. This arrest likely occurs to prevent further development of certain chromosomally abnormal embryos and/or embryos that fail to activate embryonic genome around the 4- to 8-cell stage [12]. Of note, this early developmental block does not seem to depend on sex of embryos. Actually, a non-significant sex ratio of 1.05 (86/82) has been evidenced in arrested embryos that do not pass the 8-cell stage after IVF [13]. And secondly, as shown in Table 1, the sex ratio of both ICSI 8-cell embryos (1.09) and day-5 blastocysts (0.98) is close to unity suggesting that further developmental arrest after the 8-cell stage is not sex dependent. Indeed, the developmental potential of ICSI 8-cell embryos towards the early, full or hatched-blastocyst stage on day 5 is similar between male (23.1%, 110/475) and female (21.6%, 88/408) embryos [14]. Consequently, we can assume that the sex ratio at both fertilization and implantation is between 1.29 and 1.50 in IVF cycles (the sex ratio of cleavage-stage embryos) and 1.07, 1185/1110, in ICSI cycles (this estimate results from combining sex ratios of cleavage-stage and blastocyst-stage ICSI embryos; see Table 1). We should note that the estimates of sex ratios at fertilization and implantation in IVF cycles are not robust due to the relative small number of embryos analyzed (n = 117) and the bias that may be introduced by inferring sex ratios at fertilization and implantation from data of cleavage-stage embryos. We should bear in mind the work by Fiala [15] pointing out that the sex ratio of surviving offspring cannot correctly be used to estimate the primary sex ratio because of the potential sex differential of mortality. Unfortunately, obvious ethical reasons prevent assessing directly sex ratios at fertilization and implantation in human beings.

The second option, i.e., the sperm preparation technique used in IVF may increase the proportion of Y-bearing spermatozoa, can be also rejected. In fact, it has been shown that the swim-up technique does not selectively enrich either X- or Y-bearing spermatozoa [16-18]. As mentioned above, only in heavy smoking men swim-up technique may increase the proportion of X-bearing (instead of Y-bearing) spermatozoa resulting in higher incidence of female embryos after ICSI [4]. Moreover, it is known that the three-layer discontinuous Percoll density gradient selects spermatozoa with better motion characteristics, more hyperactivation, and improved longevity compared with direct swim-up [19]. However, studies aimed to ascertain the efficiency of discontinuous Percoll density gradient centrifugation in sperm sorting show either no significant effect on X:Y ratio of spermatozoa or even an enrichment of X-bearing spermatozoa that seems to be insufficient for clinical use in pre-conceptional sex selection (for references, see Lin et al. [20]).

The third and fourth possibilities, i.e., oocytes may be more susceptible to fertilization by Y-bearing spermatozoa and/or Y-bearing spermatozoa may have higher fertilization ability, are more likely to be true. Indeed, recent evidence strongly suggests that oocytes during a critical time in folliculogenesis may change the molecular composition of the zona pellucida, e.g., a subtle variation in a sperm-binding carbohydrate on the zona-pellucida proteins induced by high levels of follicular-fluid testosterone. This molecular change may render oocytes more susceptible to fertilization by Y-bearing spermatozoa (for a review, see Grant and Chamley [21]). In addition, there are convincing data on the presence of distorter genes, expressed and translated after meiosis in round spermatids and spermatozoa, able to skew the sex ratio by affecting spermatid maturation and fertilizing ability of either X- or Y-bearing spermatozoa (for a review, see Ellis et al. [22]). This fact suggests that human spermatids and spermatozoa may “intrinsically” express distorter genes favoring spermatid maturation and fertilizing ability of Y-bearing spermatozoa.

### Implantation and early post-implantation stages before pregnancy becomes clinically recognized

Table 2 shows data retrieved from United States [23] and Australia and New Zealand [24] assisted reproductive databases. We selected these studies because they focused their analyses on large samples of ART singleton deliveries [23] or births resulting from single embryo transfers [24]. Noteworthy, Dean et al. [24] included in the calculation and analysis of sex ratio at birth only one baby from each set of multiple births. This strategy eliminated the potential bias that monozygotic twins may introduce in the calculation of sex ratio at birth. These data indicate that extended embryo culture to the blastocyst stage is associated with higher sex ratio at birth compared with shorter embryo culture to the 4- or 8-cell stage (1.25 versus 1.03 in IVF cycles and 1.04 versus 0.95 in ICSI cycles). Moreover, sex ratio at birth is lower in ICSI cycles than in IVF cycles after cleavage- (0.95 versus 1.03) and blastocyst-stage (1.04 versus 1.25) transfer. These results are qualitatively consistent with a previous systematic review and meta-analysis [25] and previous studies [26-29] not included in Table 2 because they did not provide the appropriate information and/or did not control for the potential bias associated with monozygotic twining.

The higher sex ratio at birth evidenced after blastocyst-stage transfer is not likely a consequence of embryo grading systems that prioritize male embryos for transfer as suggested by Alfarawati et al. [30]. Indeed, despite an early study [31] reported that male IVF human preimplantation embryos display increased number of cells and metabolic activity than female embryos, strong evidence shows that human preimplantation male embryos do not cleave faster [32-34], exhibit better morphology [32] and/or have higher developmental potential [13,14] than female embryos. This fact suggests that the human endometrium does not select the sex of implanting embryos as previously hypothesized by Krackow [35] and Tarín et al. [36], or evidenced in mouse embryos displaying sex-dimorphic developmental rates [37,38]. Instead, we propose that the higher secondary sex ratio found after blastocyst-stage transfer may be due to preferential female mortality at early post-implantation stages induced, at least in part, by abnormal inactivation of one of the two X-chromosomes (mechanism of dosage compensation).

#### XCI in the mouse model

Two recent reviews by Lee and Bartolomei [39] and Lessing et al. [40] show that in the mouse XCI begins during the first meiotic prophase of spermatogenesis. After completion of meiosis, the X-chromosome does not completely reactivate. Indeed, 85% of X-linked genes remain suppressed through spermiogenesis. Thus, the paternal X-chromosome is passed onto the next generation in a partially inactivated state. At the 2-cell stage, transcription of repetitive elements on the paternal X-chromosome is already suppressed, but transcription of X-linked coding genes is active. At the 8-16-cell stage (morula stage), the silencing of paternal coding genes is initiated, and is completed at the blastocyst stage or later. Gene silencing absolutely requires *cis* accumulation of a long non-coding *Xist* RNA that coats the X-chromosome and binds Polycomb repressive complex 2 (PRC2), the epigenetic complex responsible for trimethylation of histone H3 on lysine 27 (H3K27me3), a repressive epigenetic mark that leads to further silencing of the paternal X-chromosome. This is not the case for silencing repetitive elements on the paternal X-chromosome. By the 2-cell stage, although *Xist* RNA is present, repetitive elements are silenced in a *Xist* independent manner. The maternal X-chromosome is protected from inactivation through expression of *Xist*’s antisense repressor, *Tsix*.

As paternal XCI is heritable through mitosis, the paternal X-chromosome remains inactivated in both the trophectoderm and the primitive endoderm (hypoblast). In contrast, in the inner cell mass (ICM), the paternal X-chromosome undergoes reactivation. We should bear in mind that the trophectoderm gives rise to the fetal portion of the placenta; the primitive endoderm originates the parietal endoderm that contributes to the parietal yolk sac, and the visceral endoderm that contributes to the visceral and intraplacental yolk sacs; and the ICM gives rise to the epiblast lineage that develops into the embryo proper and the extra-embryonic mesoderm that forms the allantois and the mesodermal components of the visceral yolk sac, amnion and chorion (for reviews, see Hemberger [41] and Gasperowicz and Natale [42]).

Starting from the period shortly after implantation, X-chromosomes in the epiblast experience random inactivation, i.e., the maternal X-chromosome is inactive in some cells whereas the paternal X-chromosome is inactive in other cells. Paternal X-chromosome reactivation also occurs in primordial germ cells in preparation for equal segregation during meiosis (for reviews, see Lee and Bartolomei [39] and Lessing et al. [40]).

#### XCI in humans

Unlike in mice, *XIST* expression is not imprinted in humans. *XIST* expression is detected from the 4- to 8-cell stage at the onset of genomic activation [43]. Both ICM and trophectoderm show similar *XIST* RNA accumulation in their cells. However, *XIST* upregulation does not result in immediate onset of chromosome-wide XCI even in late (day-7) blastocysts [44]. Recently, Teklenburg et al. [45] using an in-vitro model for human implantation observed that implanting day-8 female embryos had distinct H3K27me3 foci (presumably on the inactive X-chromosome) localized to the trophectoderm lineages and to lesser extend the hypoblast lineages, but not in epiblast cells. These findings indicate that in the majority of the cells of human embryos, silencing of the X-chromosome may occur after the embryo has implanted. This conclusion contradicts data from another study reporting that *XIST* RNA accumulation is associated with transcriptional silencing of the *XIST*-coated chromosomal region as early as the morula and the blastocyst stage [43]. Discrepancies between studies may be explained by differences in efficiency of the immunofluorescence/FISH technique in detecting biallelic RNA signals and/or the use of different culture conditions (cited by Okamoto et al. [44]).

Early studies suggested the occurrence of paternal XCI in the fetal side of placentae. These studies analyzed the expression pattern of single X-linked genes. However, other studies using more robust analyses of multiple allele-specific gene expression along the X-chromosome support the notion that XCI in human placentae is random (for a review, see Lee and Bartolomei [39]). Similarly, it is generally accepted that X-chromosomes in the ICM lineage undergo random inactivation (for a review, see Migeon [46]). Notwithstanding, a recent study has shown that the bell-shaped distribution (centered around 50%) of X-inactivation patterns in large populations of normal women fits better a three-allele model of genetically influenced XCI than models of completely random inactivation [47].

We should emphasize that not all the X-linked genes are silenced at X-inactivation. In humans, more than 15% of genes carried on the X-chromosome appear to escape inactivation (for a review, see Brown and Greally [48]). Consequently, differences in gene dosage may explain differences between men and women in developmental programming and disease susceptibility and behavior (for a review, see Aiken and Ozanne [49]). Moreover, although XCI in human epiblast, hypoblast and trophectoderm cells likely occurs during/after implantation (see above), the silencing process may be disrupted during preimplantation stages by any factor that interferes with DNA methylation, histone deacetylation or chromatin modifications. The resulting increased or decreased X-linked gene expression may prevent embryos to either implant or develop normally after implantation (for reviews, see Hemberger [50] and Schulz and Heard [51]). We propose that extended exposure of preimplantation female embryos to suboptimal (non-physiological) culture systems may be “one” of these factors.

#### Precocious XCI in human embryonic stem cells (hESCs)

It has been reported [52] that the conventional method of hESCs (pluripotent cell types derived from the ICM of human blastocysts) derivation and maintenance under atmospheric O_2_ conditions (≈20% O_2_) as well as exposure to other cellular stresses such as harsh freeze-thaw cycles, inhibition of the proteosome, HSP90, gamma-glutamylcysteine synthetase, and treatment with organic peroxide, induces precocious random XCI prior to cellular differentiation. This precocious XCI is associated with either *XIST* expression in most or all cells, or the absence of *XIST* expression and failure to reactive *XIST* expression upon differentiation. This response differs from that found under 5% O_2_ concentration. In this case, the precocious random XCI in hESCs is prevented, being both X-chromosomes active. Furthermore, hESCs exhibit no *XIST* expression and retain the ability to activate *XIST* gene expression upon differentiation.

It is worth mentioning that nowadays in many IVF laboratories gametes and embryos are still exposed to non-physiological culture systems including atmospheric O_2_ concentrations despite data from a systematic review and meta-analysis [53] suggest that embryo culture to the blastocyst stage under low-oxygen concentration (≈5%) versus high-oxygen atmospheric concentration yields higher live birth rates. Thus, it can be inferred that embryos cultured to the blastocyst stage (embryo transfer on day 5 or 6) under non-physiological environments including atmospheric O_2_ concentrations are more susceptible to undergo epigenetic changes than embryos cultured for shorter periods of time (embryo transfer on ≤ day 3). Like hESCs, these epigenetic changes may interfere with the normal process of *XIST* expression and XCI in female embryos. Importantly, in-vitro-produced preimplantation bovine embryos display higher levels of *XIST* expression than their in-vivo counterparts, suggesting that in-vitro-culture conditions induce premature XCI [54].

We should stress that in the subgroup of hESC lines displaying precocious XCI and *XIST* expression in most or all cells when exposed to atmospheric O_2_ conditions [52], *XIST* expression was unstable and subject to stable epigenetic silencing by DNA methylation. The resulting inhibition of *XIST* expression reactivated a portion of X-linked alleles on the inactive X-chromosome (12% of X-linked promoter CpG islands became hypomethylated) [55]. Such a reactivation resulted in over-expression of X-linked genes, event that if took place in implanting female blastocysts may produce severe abnormalities in embryonic and extra-embryonic (trophoblast) tissues and early embryonic death (for a review, see Schulz and Heard [51]).

#### Data supporting and refuting the hypothesis of occurrence of precocious XCI in human female embryos

The hypothesis of occurrence of precocious XCI in female embryos exposed for extended periods of time to non-physiological culture systems is questioned by (i) the absence of significant differences in miscarriage percentage per couple after cleavage- (8.0%, 86/1069) and blastocyst-stage (9.2%, 97/1058) transfer; and (ii) the higher live-birth percentage per couple after blastocyst-stage transfer (38.9%, 292/751, versus 31.2%, 237/759, after cleavage-stage transfer) (for a systematic review and meta-analysis, see Glujovsky et al. [56]). As a matter of fact, we should expect higher miscarriage percentages and lower live-birth percentages after blastocyst-stage transfer if a given percentage of female embryos undergoes precocious XCI. However, it is generally thought that extended culture selects those embryos that have proven ability to survive and develop to an advanced stage in vitro [although a wide range of blastulation rates has been reported (from 28% to 97%), on average only 46.8% of embryos reach the blastocyst stage (for a systematic review and meta-analysis, see Glujovsky et al. [56])]. This fact together with the presence of an uterine environment that likely is more synchronized compared with cleavage-stage transfers ( [57]; for a review, see Bourgain and Devroey [58]) may contribute to the similar miscarriage rates and higher live-birth percentages reported after blastocyst-stage transfer compared with cleavage-stage transfer.

In addition, the incidence of female losses (presumably caused by precocious XCI) is likely higher at early stages of pregnancy before women are aware that they are pregnant than after pregnancy has been clinically recognized (note that early pregnancy losses are not taken into account when analyzing miscarriage percentages). In this context, we should mention that blastocyst-stage transfer is associated with higher percentage of biochemical pregnancy losses per embryo transfer (14.1%, 108/767) [59] than cleavage-stage transfer (8.2%, 154/1888) [60].

### Late post-implantation stages after pregnancy becomes clinically recognized

Shortly after pregnancy becomes clinically recognized, females keep displaying a developmental disadvantage compared with males. This disadvantage subsequently vanishes as gestational age increases. In particular, by combining the data reported by Eiben et al. [61] and Yusuf and Naeem [62], sex ratios of chromosomally normal abortions increase from 0.46, 67/147, at 5–9 weeks of pregnancy to 0.79, 137/173, at 10–13 weeks and 1.02, 269/263, at ≥ 13 weeks. A concomitant increase in natural selection against males with gestational age is also evidenced in chorionic villus sampling and amniocentesis material from control pregnant women. In these ongoing pregnancies, sex ratios significantly decrease from 1.28, 791/618, at < 16 weeks of pregnancy to 1.06, 25433/23994, at ≥ 16 weeks [63]. We should bear in mind that human males and females develop at different rates in uterus (and postnatally until the postpubertal stage). Thus, male fetuses have a greater effective exposure to a given insult than female fetuses that undergo fewer cell cycles during the same period of exposure (for a review, see Aiken and Ozanne [49]).

### Birth

Table 2 shows that, compared with the estimated sex ratio at implantation (1.29 to 1.50 in IVF cycles and 1.07 in ICSI cycles), the sex ratio at birth is lower in IVF cycles (1.03 and 1.25 after cleavage- and blastocyst-stage transfer, respectively) but similar and closer to unity in ICSI cycles (0.95 and 1.04 after cleavage- and blastocyst-stage transfer, respectively). Note that we should expect lower sex ratios at birth than at implantation if male mortality during pregnancy surpasses female losses. On the contrary, we should expect sex ratios at birth similar to or even higher than sex ratios at implantation if female mortality is comparable or exceeds male mortality.

We should stress that sex ratios at birth are closer to sex ratios at implantation after blastocyst-stage-transfer than after cleavage-stage-transfer. This fact is in consonance with the hypothesis of occurrence of precocious XCI in female embryos cultured in vitro to the blastocyst stage. Likewise, sex ratios at birth are nearer to sex ratios at implantation in ICSI than in IVF cycles. In this context, we should mention the study by Dumoulin et al. [64] reporting decreased number of trophectoderm cells in ICSI female blastocysts compared with ICSI male blastocysts (this effect was not observed in IVF blastocysts). As the trophectoderm lineage gives rise to the fetal portion of the placenta, ICSI female blastocysts may exhibit higher incidence of abnormal trophoblast function and decreased potential for implantation and further development compared with ICSI male blastocysts.

## Concluding remarks

Data from genetically diagnosed preimplantation embryos suggest that the sex ratio at both fertilization and implantation is between 1.29 and 1.50 in IVF cycles and 1.07 in ICSI cycles. Embryo exposure to culture media for extended periods of time to the blastocyst stage under non-physiological conditions (e.g., under atmospheric O_2_ conditions) may induce precocious XCI in female embryos. Such a precocious XCI together with ICSI-induced decrease in number of trophectoderm cells in female blastocysts may account for preferential female mortality at early post-implantation stages and thereby variations in sex ratios at birth in ART cycles. In particular, in IVF cycles the early developmental disadvantage of females would be surpassed by the higher mortality rates of males later in pregnancy resulting in lower sex ratios at birth than at implantation. In contrast, in ICSI cycles early female mortality would be comparable to later male mortality affording similar sex ratios at birth and implantation. Blastocyst transfer in both IVF and ICSI cycles would be associated with higher post-implantation female mortality than cleavage-stage transfer. Consequently, sex ratios at birth would be closer to sex ratios at implantation after blastocyst transfer than after cleavage-stage transfer.

The hypothesis of precocious XCI may be extended to natural cycles to explain, at least in part, some biases of sex ratio at birth observed in human populations/families (for reviews, see James [65,66]). In particular, disturbances of XCI may be induced by biological (e.g., gametes from reproductive-old women/men and pre- or post-ovulation/ejaculation aged gametes) or environmental (e.g., maternal exposure to nutritional deficits/excesses, physical/psychological/social stresses, medications, social drugs, radiations, environmental pollutants and chemotherapy agents) factors. Certainly, this is a research area that needs further attention.

## Abbreviations

5mC: Fifth carbon of the cytosine base; ART: Assisted reproduction treatment; EBSS: Earle’s balanced salt solution; FISH: Fluorescent in situ hybridization; H3K27me3: Histone H3 on lysine 27; hESCs: Human embryonic stem cells; ICSI: Intracytoplasmic sperm injection; IVF: In-vitro fertilization; PRC2: Polycomb repressive complex 2; XCI: X-chromosome inactivation.

## Competing interests

The authors declare that they have no competing interests.

## Authors’ contributions

JJT was involved in the conception and design of the study, the acquisition, analysis and interpretation of data and drafting of the article. MAGP, CH and AC were involved in the analysis and interpretation of data, and revising the article critically for important intellectual content. All authors read and approved the final manuscript.
